# Swab Materials and *Bacillus anthracis* Spore Recovery from Nonporous Surfaces

**DOI:** 10.3201/eid1006.030716

**Published:** 2004-06

**Authors:** Laura Rose, Bette Jensen, Alicia Peterson, Shailen N. Banerjee, Matthew J. Arduino

**Affiliations:** * Centers for Disease Control and Prevention, Atlanta, Georgia, USA

**Keywords:** Swab, swab efficiency, surface sampling, *Bacillus anthracis*, spores, Anthrax

## Abstract

Four swab materials were evaluated for their efficiency in recovery of *Bacillus anthracis* spores from steel coupons. Cotton, macrofoam, polyester, and rayon swabs were used to sample coupons inoculated with a spore suspension of known concentration. Three methods of processing for the removal of spores from the swabs (vortexing, sonication, or minimal agitation) and two swab preparations (premoistened and dry) were evaluated. Results indicated that premoistened swabs were more efficient at recovering spores than dry swabs (14.3% vs. 4.4%). Vortexing swabs for 2 min during processing resulted in superior extraction of spores when compared to sonicating them for 12 min or subjecting them to minimal agitation. Premoistened macrofoam and cotton swabs that were vortexed during processing recovered the greatest proportions of spores with a mean recovery of 43.6% (standard deviation [SD] 11.1%) and 41.7% (SD 14.6%), respectively. Premoistened and vortexed polyester and rayon swabs were less efficient, at 9.9% (SD 3.8%) and 11.5% (SD 7.9%), respectively.

The Centers for Disease Control and Prevention (CDC), along with its partners in public health, law enforcement, environmental protection, defense, and the U.S. Postal Service, has been investigating a series of bioterrorism-related anthrax deaths and illnesses that occurred from October to December 2001. As of January 2002, 22 cases of confirmed or suspected cutaneous or inhalation anthrax were identified (*1*). Twenty of these cases were associated, or were likely to have been associated with, materials containing *Bacillus anthracis* spores that were delivered through the U.S. Postal Service. The source of the infection remains unknown for the other two cases. During the investigation, thousands of swabs, wipes, and high-efficiency particulate air (HEPA) filter sock samples were collected. A review of the sampling data in one publication suggests that HEPA socks and wipes were superior to swabs for recovery of *B. anthracis* spores (*2*). The above-mentioned study was conducted within the contaminated Brentwood Mail Processing and Distribution Center in Washington D.C. The comparisons were considered semiquantitative in that sampling sites were chosen to be directly adjacent and the distributions of spores were assumed to be similar, but the initial inoculum was unknown.

Originally, the swab-rinse method was developed to assess bacterial contamination of food utensils (*3**–**7*). This method was modified by the National Aeronautics and Space Agency (NASA) for environmental sampling of spacecraft and equipment (*8**–**11*). Historically, the number of organisms recovered from swabs used for environmental sampling has shown a poor correlation with the amount of microbial contamination on surfaces (*3**,**12**–**14*). Several factors can contribute to this poor correlation, including differences in materials used (e.g., cotton, polyester, rayon, calcium alginate) (*3**,**13**,**15**–**17*), the organisms targeted for culture (*3**,**16**,**17*), variations in surface (10), and differences in the personnel who are collecting and processing samples (*3**,**13**,**18**,**19*).

In this study, the recovery efficiencies of four swab materials, both dry and premoistened, were compared, and different methods for swab processing were assessed for the recovery of known quantities of *B. anthracis* spores from a nonporous stainless steel surface.

## Materials and Methods

### Spore Preparation

The veterinary vaccine strain of *B. anthracis* Sterne 34F2 (Colorado Serum, Denver, CO) was grown in Leighton–Doi liquid medium (*20*) for 7 days at 36°C. The cells were checked for sporulation by microscopic examination of a slide preparation stained with malachite green (Fisher Scientific, Springfield, NJ), then harvested by centrifugation at 5,000 x *g* for 15 min and washed 3 times in sterile, ultrapure reverse osmosis (RO) water. The spores were purified by centrifugation through 58% Hypaque 76 (NYCOMED, Inc., Princeton, NJ) at 7000 x *g*, followed by three additional washes in sterile RO water. The spores were pelleted by centrifugation one final time, then resuspended in 50% ethanol. This stock spore suspension was stored at 4°C.

### Swab Description

Four types of swabs were evaluated in this study: cotton (Baxter Healthcare Corp., Deerfield, IL cat #A5002-5), polyester (Falcon, Becton Dickinson Microbiology Systems, Sparks, MD, cat #220690), rayon (Cole Parmer, Vernon Hills, IL, cat #14001-55), and macrofoam (VWR, Suwanee, GA, cat #10812-046). Surface characteristics were visualized by environmental scanning electron microscopy (SEM).

### Direct Inoculation

The stock spore suspension was added to Butterfield Buffer (BB) (3 mmol/L KH_2_PO_4_, pH 7.2; Becton Dickinson Microbiology Systems) to attain 0.5 McFarland standard containing 10^6^ CFU of spores/mL with a Microscan turbidity meter (Dade Behring, West Sacramento, CA). This suspension was diluted 1:10 in BB, and the swabs were inoculated directly with 100 µL of this dilution to compare the ability of each material to retain spores. Swabs were placed immediately into tubes containing 5 mL phosphate-buffered saline (pH 7.2) containing 0.04% Tween 80 (PBST) and vortexed at high speed for 2 min in 10-s bursts. Serial dilutions were performed (10^–1^–10^–5^) in BB, and 100 µL from each tube was spread onto each of three plates of Trypticase soy agar containing 5% sheep blood (TSAB, Becton Dickinson Microbiology Systems). Plates were incubated at 36°C overnight, and colonies were counted the next day.

### Preparation of Coupons

Stainless steel coupons (2 x 2 inches) were cut from a sheet of S-180 grade, T-304 stainless steel (Stewart Stainless Supply, Inc., Suwanee, GA) and were used as test surfaces. This grade of stainless steel is commonly used in food service settings (J. Willingham, Stewart Stainless Supply, Inc., pers. comm.). The stainless steel was previously characterized for roughness by using a profilometer (Tencor AS500 profilometer, KAL-Tencor, San Jose, CA) and for contact angle (hydrophobicity) with a goniometer (Ramé-Hart, model number 100-00, Ramé-Hart, Inc, Mountain Lakes, NJ) (*21*). Surface characteristics had been visualized previously by environmental SEM (Phillips SL30 ESEM, FEI Co., Hillsboro, OR) (*21*).

Each coupon was washed with nonbactericidal detergent (Versa-Clean, Fisher Scientific, Pittsburgh, PA); rinsed with ultrapure, RO water, air dried, placed into 10x100-mm glass petri dishes, and sterilized in an autoclave. A spore preparation was adjusted to a 0.5 McFarland standard with a Microscan turbidity meter, resulting in a 1 x 10^6^ CFU spores/mL suspension. This suspension was diluted 1:10 in 95% ethanol and vortexed at high speed for 1 min. A 0.5-mL aliquot was placed on the coupon with a repeat pipettor, then evenly spread over the surfaces of each of the stainless steel coupons with the side of a sterile disposable pipette tip. The lids of the petri dishes were closed, and the dishes with test coupons were placed in a biological safety cabinet and allowed to dry overnight. The coupons were then sampled with swabs.

### Sampling

For each material, 70 spore-laden coupons were used: 10 controls, 30 sampled with dry swabs, and 30 sampled with swabs premoistened with PBST. If premoistened, swabs were dipped in a tube containing PBST, then pressed against the side of the tube to express excess liquid. Swabs were swiped across each coupon methodically in a horizontal, then vertical, and then diagonal direction several times. During sampling, care was taken to sample up to, but not over, the edge of the coupon. The swabs were rolled to expose unused sides as they were moved across the surface of the coupon.

After sampling, swabs were placed into tubes containing 5 mL of PBST. From the 60 swabs of each material that were used for sampling, 10 premoistened and 10 dry swabs were subjected to minimal agitation, 10 premoistened and 10 dry swabs were vortexed for 2 min in 10-s bursts, and 10 premoistened and 10 dry swabs were placed into a Branson 42 kHz (100 W) ultrasonic bath (Branson Instruments, Danbury, CT) and sonicated for 12 min. Serial dilutions were performed (10^–1^–10^–5^) in BB, and 100 µL from each dilution tube was spread on TSAB plates in duplicate. Plates were incubated at 36°C overnight, and colonies were counted the next day.

Ten control coupons were processed as follows: each coupon was aseptically transferred to a 600-mL beaker and covered with 20 mL of PBST, sonicated for 12 min, and then scraped with a sterile cell scraper (Fisher Scientific, cat # 07-200-365) for 1 min to remove the spores. Two mL from the 600-mL beaker was plated directly onto TSAB plates (500 µL to each of four plates). Serial dilutions were performed (10^–1^–10^–3^) in BB, and 100 µL from each dilution tube was spread on TSAB plates in duplicate. Plates were incubated at 36°C overnight, colonies were counted the next day, and the number of CFUs recorded.

### Analysis and Statistics

Ten coupons were used for each swab material, swab preparation, and processing protocol to be evaluated. This procedure allowed us to identify significant differences in the sample means (CFUs) of >12% with 80% power. Mean CFUs were determined for each dilution, and the total number of organisms per coupon was calculated by multiplying by the dilution factors. Percent recovery efficiencies (%RE) were calculated by using the following equation: %RE = (Σ [N_SW_/N_0_]/n) x 100, where N_0_ is the number of CFUs from the control surfaces, N_SW_ is the number of CFUs from the swab material, and n is the sample size. Effects of swab preparation and processing protocol (combined as recovery method) and swab materials and their interactions were analyzed with general linear model procedure for analysis of variance of unbalanced data. Pairwise comparison of appropriate treatment means was done by Student *t* test and also by Bonferroni adjustment for multiple comparisons (*22*).

## Results

### Directly Inoculated Swabs versus Surface-Sampled Swabs

Scanning electron micrographs of the swab materials used in this study are shown in the Figure. At the scale provided by micrographs, three materials (polyester, rayon, and cotton) appear to have fibers similar in size and density, though the polyester has more spaces closed by irregularly shaped fibers. The macrofoam appears to have a more open structure than the other three materials. When swabs were inoculated directly with the spore suspension, then processed with vortexing, all swab materials tested released significantly higher percentages of spores than were recovered by swabs that sampled spores from the stainless steel surfaces (Table 1). No significant differences were observed between cotton, macrofoam, and rayon in their abilities to release spores (p > 0.05) when directly inoculated. Cotton, macrofoam, and rayon released 93.9%, 93.4%, and 91.7% of spores inoculated onto them, respectively. The polyester swab released a significantly lower percentage than the other three materials (83.8%, p < 0.01).

**Figure:**
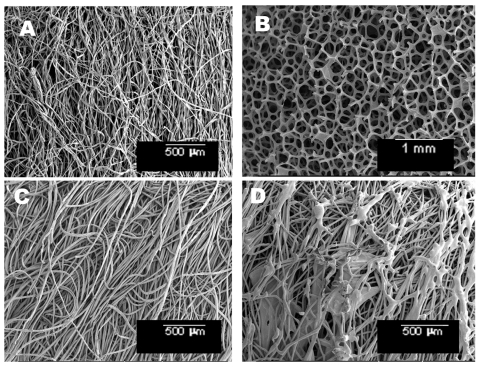
Environmental scanning electron micrographs of swab material: cotton (A), macrofoam (B), rayon (C), polyester (D).

**Table 1:** Percentage of spores recovered from premoistened, directly inoculated, and vortexed swabs^a^

Swab material	Mean	Median	SD	Range^b^	95% CI^c^
Cotton	93.9	93.1	10.1	72.5–112.9	87.7–100.2
Macrofoam	93.4	96.6	10.8	73.4–107.7	86.6–100.1
Polyester	83.8	81.9	7.2	73.3–98.2	79.4–88.3
Rayon	91.7	92.6	6.3	79.8–100.7	87.8–95.5

^a^N = 10. ^b^Percentages calculated relative to mean of control tests, allowing maximum to be >100%. ^c^CI, confidence interval.

### Dry versus Moist Swabs

For each material tested, premoistened swabs were more efficient than dry swabs at recovering spores from the stainless steel coupons. Results of unadjusted *t* tests show that recovery of spores from all compared materials (Table 2) is significantly improved by premoistening the swabs, regardless of which processing protocol is used (p < 0.05). However, when the multiple comparison adjustment was applied, the efficiency of polyester and rayon swabs do not appear to be significantly improved by premoistening (p = 1.0), regardless of processing method. When no extraction was performed during laboratory processing of the swabs, no significant differences were found between spore recovery with dry and premoistened swabs of any material (p = 1.0) (Table 2). When sonication was used as the extraction method during laboratory processing of swabs, no differences were seen between spore recovery with dry and premoistened swabs of any material (p = 1.0) (Table 2). Spore recovery with vortexed cotton and macrofoam swabs improved significantly (p < 0.01) when swabs were premoistened. This combination of materials and processing method provided the highest percentage of spores recovered.

**Table 2:** Comparison of spore recovery efficiencies by swab preparation, material, and recovery methods

Recovery method	Mean percentage recovery from 2x2-inch steel coupon (SD), N = 10				
	All swabs	Cotton	Macrofoam	Polyester	Rayon
All					
Dry	4.4 (4.7)	5.1 (3.9)	8.4 (6.2)	1.2 (1.0)	3.0 (2.2)
Premoistened	14.3 (14.9)	20.0 (18.1)	22.5 (17.5)	7.7 (5.3)	7.0 (6.8)
p^a^	< 0.01	< 0.01	< 0.01	1.0	1.0
Extraction^b^					
Dry	6.5 (4.4)	7.5 (2.3)	12.3 (3.2)	1.7 (0.8)	4.4 (1.0)
Premoistened	19.7 (15.5)	27.7 (17.7)	30.7 (15.9)	10.6 (4.1)	10.0 (6.4)
p^a^	< 0.01	< 0.01	< 0.01	1.0	1.0
No extraction					
Dry	0.4 (0.7)	0.5 (0.4)	0.7 (1.1)	0.1 (0.2)	0.1 (0.2)
Premoistened	3.5 (3.1)	4.7 (2.2)	6.3 (3.9)	2.0 (1.0)	1.0 (0.8)
p^a^	1.0	1.0	1.0	1.0	1.0
Vortex					
Dry	6.6 (4.2)	8.0 (1.4)	11.9 (3.1)	2.1 (0.9)	4.4 (1.0)
Premoistened	26.7 (18.9)	41.7 (14.6)	43.6 (11.1)	9.9 (3.8)	11.5 (7.9)
p^a^	< 0.01	< 0.01	< 0.01	1.0	1.0
Sonication					
Dry	6.4 (4.8)	6.9 (3.0)	12.7 (3.4)	1.4 (0.5)	4.5 (1.0)
Premoistened	12.7 (5.6)	13.6 (3.2)	17.7 (5.9)	11.2 (4.4)	8.5 (4.4)
p^a^	1.0	1.0	1.0	1.0	1.0

^a^Adjusted for multiple comparisons by Bonferroni correction. ^b^Vortex and sonication combined.

### Extraction versus No Extraction

When premoistened swabs were considered, an extraction method enhanced recovery for all materials when compared to processing the same materials with minimal agitation (no extraction) (Table 3). These improved recovery efficiencies were significant for all materials (p < 0.01) when an unadjusted *t* test was used for comparison, but not for polyester or rayon when the multiple comparison correction was applied (p = 1.0). When only premoistened swabs were considered, the macrofoam yield increased from 6.3% to 30.7% with extraction, and the cotton yield increased from 4.7% to 27.7% with extraction (Table 3).

**Table 3:** Percentage recovery of premoistened swabs

Recovery method	Mean	Median	SD	Range	95% CI^a^	p^b^
All swabs						
All	14.3	9.4	14.9	0.4–63.9	11.2 to 17.4	
Extraction^c^	19.7	14.4	15.5	4.8–63.9	16.3 to 23.1 2.5 to 4.4	< 0.01
No extraction	3.5	2.7	3.1	0.4–13.5		
Vortex	26.7	23.7	18.9	1.4–29.0	20.8 to 32.6 11.0 to 14.5	< 0.01
Sonication	12.7	13.0	5.6	4.8–63.9		
Cotton swabs						
All	20.0	13.3	18.1	2.6–62.5	13.5 to 26.5	
Extraction	27.7	20.0	17.7	7.3–62.5	16.7 to 38.7 3.3 to 6.1	< 0.01
No extraction	4.7	4.0	2.2	2.6–9.7		
Vortex	41.7	43.7	14.6	23.9–62.5	33.7 to 51.8 11.3 to 15.3	< 0.01
Sonication	13.6	13.3	3.2	7.3–19.5		
Macrofoam swabs						
All	22.5	16.7	17.5	1.8–63.9	16.3 to 28.8	
Extraction	30.7	29.7	15.9	7.0–63.9	20.8 to 40.5 3.9 to 8.7	< 0.01
No Extraction	6.3	6.5	3.9	1.8–13.5		
Vortex	43.6	44.9	11.1	30.4–64.0	36.8 to 50.5 14.0 to 21.3	< 0.01
Sonication	17.7	16.7	5.9	7.0–29.0		
Polyester swabs						
All	7.7	6.4	5.3	0.5–16.5	5.8 to 9.6	
Extraction	10.6	11.1	4.1	4.8–16.5	8.1 to 13.1 1.4 to 2.5	1.0
No Extraction	2.0	2.0	1.0	0.5–3.4		
Vortex	9.9	10.0	3.8	4.8–14.4	7.5 to 12.3 8.5 to 13.9	1.0
Sonication	11.2	12.3	4.4	4.8–16.5		
Rayon swabs						
All	7.0	6.0	6.8	0.4–24.0	4.6 to 9.4	
Extraction	10.0	8.1	6.4	1.4–24.1	6.0 to 14.0 0.5 to 1.4	1.0
No extraction	1.0	0.9	0.7	0.4–2.9		
Vortex	11.5	11.5	7.9	1.4–24.1	6.6 to 11.2 5.7 to 11.3	1.0
Sonication	8.5	7.5	4.4	2.5–18.9		

^a^CI, confidence interval. ^b^Adjusted for multiple comparisons by Bonferroni correction. ^c^Vortex and sonication methods combined.

### Comparison of Premoistened, Extracted Materials

If we consider only premoistened, extracted swabs, the macrofoam and cotton were the most efficient of the four materials with percentages of recovered spores of 30.7% and 27.7%, respectively, with no significant difference between them (p = 1.0). Polyester and rayon swabs (10.6% and 10.0%, respectively, Table 3) were significantly less efficient than the cotton and macrofoam swabs (cotton and macrofoam vs. polyester and rayon, unadjusted p < 0.01). However, no significant difference was found between the recovery efficiencies of rayon and polyester swabs if swabs were premoistened and extracted (p = 1.0).

### Vortex versus Sonication

Of the two extraction methods (Table 3), vortexing premoistened macrofoam and cotton swabs (43.7% and 41.7% recovery, respectively) resulted in a significantly greater recovery than did sonication of each material (17.7% and 13.6%, respectively) (p < 0.01). The differences between the two methods were not significant for polyester or rayon (p = 1.0).

## Discussion

The swab-rinse method was originally developed by Mannheimer and Ybanez in 1917 to assess the bacterial contamination of eating utensils (*5*). In 1944, the American Public Health Association included it in its recommended methods for food utensil sanitation monitoring (*23*). It is still recommended for various applications in the food industry (*18*). NASA adapted this method for spacecraft applications and developed other methods, such as a wipe-rinse and vacuum probe method, to assess organisms in outgoing spacecraft (*8**–**11**,**24*). NASA recommended that the swab not sample more than a 4-in^2^ area and that a 2-min sonication step be included during swab extraction. The American Society for Microbiology’s Clinical Microbiology Procedures Handbook also recommends that a 2x2-in area be used in environmental and medical device sampling (*16*).

The results of this study suggest the superiority of macrofoam swabs that are moistened before sampling and vortexed during processing. The findings of this study are consistent with previous work showing the overall low efficiency of using swabs for surface sampling and the low precision of the method as reflected in the wide range in recovery of spores from steel coupons. Angelotti et al. (*3*) found that cotton swabs recovered 30.4%–69.9% of *Micrococcus pyogenes* and 30.1%–43.2% of *B. globigii* (currently *B. atrophaeus*) (*25*) spores. They suggested that the variations in a controlled laboratory setting were minimal when compared to those in field applications, where factors such as variations in sampling area, sampling technique (pressure applied, speed of sampling), distribution of spores on the surface, presence of dust or soil, or physical or chemical properties of the surface could further reduce recovery. They proposed that the low precision of swabs is not only inherent in sampling, but that each step in extraction can also introduce error that contributes to the low precision (*3*). Suggested examples of processing variables include inconsistent release of spores from swabs due to variations in vortexing or sonication, pipetting errors, and colony-counting errors. Some have suggested that alginate swabs would be better for recovery of spores, since they dissolve completely in sodium hexametaphosphate and the potential for spores to be retained in the swab would be eliminated. Angelotti et al. (*3*) and Strong et al. (*26*), however, found that calcium alginate swabs were less efficient at removing spores from a surface than were cotton swabs, and may inhibit some organisms, including *B. globigii* spores.

Work by Barnes (*13*) showed that the percentage of *Bacterium* (currently *Escherichia*) *coli* and *Staphylococcus albus* recovered from a smooth drinking glass by a cotton swab varies with inoculum level. For *E. coli*, the percentage recovered was lower when the inoculum was higher (56% at 10^4^/glass and 40% at 10^5^/glass), but *S. albus* demonstrated a higher percentage recovered with a higher inoculum (38% at 10^4^/glass, and 71% at 10^5^/glass). Inherent differences likely exist in each organism’s ability to adhere to smooth glass. *B. anthracis* spore adherence properties were not explored in this study. Hucker et al. (*27*) demonstrated that recovery of microorganisms from surfaces by cotton swabs is directly proportional to the ease of wetting the surface. This work reinforces the idea that swabs should be premoistened with a solution containing a surfactant, such as Tween 80, for maximum retrieval of spores.

Sampling efficiency of cotton swabs was investigated by Buttner et al. (*28*), in which glass petri dishes were inoculated with 10^6^ *B. subtilis* subsp. niger (currently *B. atrophaeus*) (*25*) spores suspended in buffer with 0.05% Tween 20, distributed within a 5-cm^2^ area and sampled with cotton swabs. The higher mean recovery (68.6%) in this study may be attributed to the higher spore inoculum contained in a smaller surface area, reduced spore adherence to the more hydrophilic glass surface, or the spores being suspended in the buffer with a surfactant that would also reduce adhesion to the surface.

Our study found that recovery was most efficient when macrofoam or cotton swabs were moistened before sampling and subjected to vortex extraction. Puleo et al. (*24*) reported that sonication provided a better recovery of *B. subtilis* subsp. niger (currently *B. atrophaeus*) (*25*) spores than mechanical agitation from stainless steel coupons. Their study differed in that the mechanical agitation in Puleo’s study consisted of placement on a platform shaker at 270 oscillations per min for 10 min, rather than agitation by vortexing, which provides a more vigorous motion, as was done in this study. Since Puleo’s experimental methods and equipment differed from those used in this study, a comparison of results may not be valid. His study, however, does illustrate the wide variability of recovery inherent in sampling with swabs. Puleo et al. (*29*), in a separate study, also established that sonication does not affect spore viability.

When swabs were inoculated directly, approximately 84%–94% of spores were recovered, yet surface sampling in the current study yielded <50% of spore inoculum. If the swabs retain only 6.1%–16.2% of the spores, the differences in recovery efficiencies of spores from directly inoculated swabs and those used to sample spore-inoculated surfaces can be explained only by assuming a substantial number of spores remain on the stainless steel coupon. Unlike powder preparations, spores, when applied with alcohol may become fixed to the surface after evaporation of the alcohol, which may represent a challenge to their recovery; however, the method provides a standard application to enable comparison of the swab materials and processing protocols. In this evaluation, no attempt was made to measure the amount of spores remaining fixed to the coupon surface. Since a perception exists (though no supporting data could be found by the authors) that polymerase chain reaction–based methods for detecting *B. anthracis* in processed samples are hindered by the presence of cotton fibers or impurities associated with cotton swabs, it was important to find that comparable results can be obtained by using macrofoam swabs.

Though no significant differences were seen between premoistened and dry rayon or polyester swabs, regardless of the processing method (Table 2), all of these recovery efficiencies were <11.5%, and in many cases, standard deviations were high. Similarly, no significant differences were seen between extracted and nonextracted premoistened rayon and polyester swabs (Table 3). The percentage recovery efficiencies of each of these groups were small, and the standard deviations were large.

All currently available environmental sampling techniques (i.e., wipes, HEPA sock) have inherent advantages and disadvantages. Each method should be evaluated to determine the overall recovery efficiencies of the materials together with the processing protocols. With this information, incident response personnel will be better able to choose the best sampling methods needed for each surface within the contaminated area. Swabbing environmental surfaces may not be the most efficient means of recovering bacterial contamination if quantitation (i.e., estimate of magnitude) is the objective of the sampling; however, in some situations a swab sample may be the best available sampling method. We hope that this brief study will help in the choice of the best material for environmental sampling and aid in interpreting results.
